# Renal cell carcinoma of the native kidney in renal transplant recipients: case report and literature review

**DOI:** 10.3389/fonc.2025.1536411

**Published:** 2025-04-29

**Authors:** Yi Tao, Jun Wang, Yulan Peng, Jiaojiao Zhou

**Affiliations:** Department of Medical Ultrasound, West China Hospital of Sichuan University, Chengdu, China

**Keywords:** renal cell carcinoma, kidney, transplantation, ultrasound, diagnosis, case report

## Abstract

Kidney transplant recipients (KTRs) carry an elevated risk of cancer-related mortality. The cumulative incidence of *de novo* post-transplant malignancy (DPTM) reaches 10% at 10 years, with renal cell carcinoma (RCC) arising in native kidneys being the predominant urologic malignancy. This study presents three KTRs who developed native kidney RCC 6–15 years post-transplantation. Notably, Case 1 demonstrated a 14.7 cm mass at diagnosis, secondary to non-adherence to protocol-based native kidney surveillance. Histopathological confirmation of RCC was established in all cases through ISUP/WHO-graded surgical specimens and immunophenotypic profiling. KTRs exhibit elevated native kidney RCC risk, often with nonspecific clinical presentations. Our findings emphasize the critical role of systematic imaging protocols, particularly ultrasonography and contrast-enhanced ultrasound (CEUS), in early tumor detection. Implementing these strategies may improve survival and reduce disease burden in this high-risk population.

## Introduction

It has been reported that the cumulative incidence of *de novo* post-transplant malignancy (DPTM) after kidney transplantation ranges from 8% to 15%, with a median onset time of approximately 6.5 years (1). The urinary system is the most frequently affected organ system, accounting for 32.1% of cases. Among these, renal cell carcinoma (RCC) represents the most common urinary malignancy in kidney transplant recipients (KTRs) (2–4). This phenomenon is not only linked to the physiological alterations induced by transplantation but also closely associated with chronic immunosuppression from post-operative regimens.

Approximately 80.4% of RCC cases in KTRs originate in the native kidneys (4–7). End-Stage Renal Disease (ESRD) and acquired cystic kidney disease (ACKD, defined as a progressive polycystic kidney condition secondary to ESRD or prolonged dialysis) are strongly correlated with the development of primary RCC post-transplantation. Notably, clear cell renal cell carcinoma (CCRCC) and papillary renal cell carcinoma (PRCC) predominate as histologic subtypes, frequently exhibiting bilateral and multifocal presentation (8, 9).

This study presents three cases of primary RCC in native kidneys of KTRs. In Case 1, delayed tumor detection due to irregular imaging surveillance resulted in diagnosis at an advanced stage. We describe the clinical manifestations, imaging features, and pathologic findings in these patients. Furthermore, by integrating existing literature on clinical management of KTRs, we comprehensively analyze the epidemiology, early diagnostic approaches, and therapeutic strategies for native kidney RCC following transplantation. From an imaging perspective, this review aims to refine tumor surveillance protocols for KTRs, promote timely detection, and ultimately improve long-term outcomes and quality of life.

## Case presentation

### Case 1

#### Chief complaints

A 53-year-old female patient, 13-year post-renal transplantation, presented to our hospital with a 4-month history of elevated creatinine levels.

#### History of present illness

The patient underwent allogeneic kidney transplantation at our hospital 13 years ago for ESRD.Four months prior, serum creatinine levels of approximately 180 μmol/L were detected at an external facility. The patient denied symptoms such as fever, cough, or abdominal pain and received no specific treatment. Ten days prior to admission, she developed bilateral lower extremity edema, reduced urine output (approximately 1/3 of baseline), increased nocturia, urinary frequency, and urgency. Outpatient laboratory studies revealed: serum creatinine: 186 μmol/L (reference range: 48–79 μmol/L); uric acid: 461 μmol/L (reference range: 160–380 μmol/L); urinalysis: nitrite positive (+), bacteria >10,000/μL (reference: <230/μL), WBC 15/HPF (reference: 0–5/HPF).

The patient was admitted to the hospital due to renal transplant dysfunction and urinary tract infection. Preoperative ultrasound-guided percutaneous renal biopsy of the transplanted kidney was performed. Pathological examination revealed no glomeruli in the light microscopy samples. However, eight glomeruli were identified upon PASM staining of frozen sections, with most exhibiting glomerulosclerosis. Additionally, moderate to severe chronic tubular-interstitial damage was observed. No definitive signs of rejection were noted.

#### Physical examination

The patient had no costovertebral angle tenderness, flank tenderness, or ureteral tenderness bilaterally.

#### Imaging examinations

##### Ultrasound examinations

**Figure 1:**
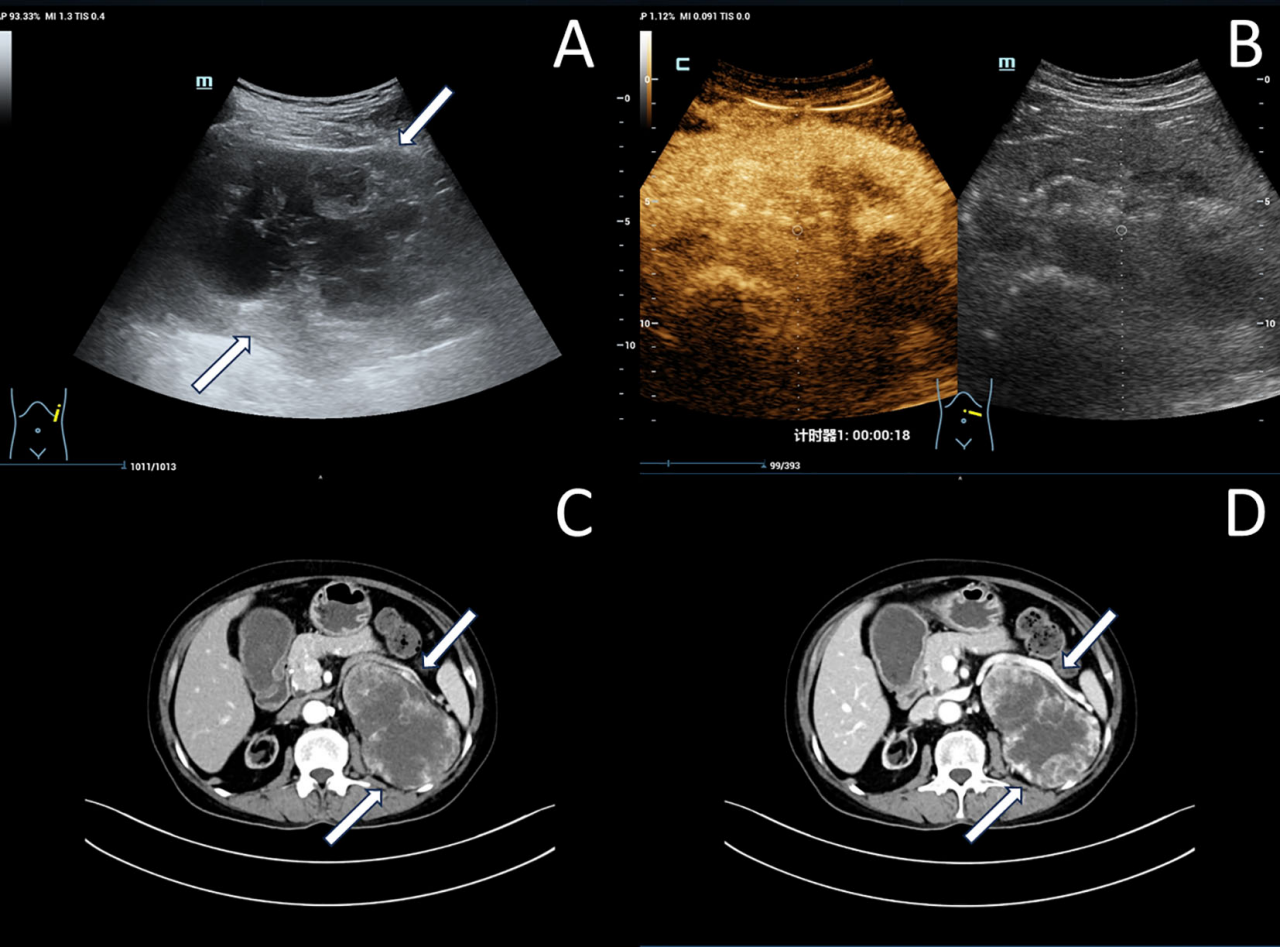
Imaging of left native renal cell carcinoma (Case 1): **(A)** Grayscale ultrasonography reveals a large cystic-solid mass in the left native renal region (white arrow). **(B)** Contrast-enhanced ultrasonography demonstrates heterogeneous hyperenhancement within the mass. **(C, D)** Enhanced computed tomography scan shows heterogeneous hyperenhancement within the mass (white arrow).

By conventional grayscale ultrasound, the contour of the left native kidney was poorly displayed, and a mixed cystic-solid echogenic mass measuring approximately 14.7×7.6×9.5 cm was detected in the region of left native kidney and the left margin of the spinal dolichoectasia (**Figure 1A**), with unclear borders and irregular morphology.

Moreover, the patient underwent a contrast-enhanced ultrasound (CEUS) examination with a bolus injection of 2mL of contrast agent SonoVue (Bracco, Milan, Italy) followed by 3 mL of saline through the antecubital vein. The solid component of the left renal cystic-solid mixed echogenic mass shows rapid heterogeneous enhancement, with no enhancement of the anechoic areas of the mass. The enhancement pattern of the mass was fast in and out, and RCC was considered possible in the ultrasound diagnosis. (**Figure 1B**).

##### Enhanced computed tomography (CT) examination:

The patient then underwent an enhanced CT examination, and the images showed a localized shadow of a huge mass in the left kidney, with a cross-section of approximately 11.2×7.7 cm, unclear boundaries, and marked enhancement of the solid component, with the left kidney being pushed and displaced (**Figures 1C, D**).

#### Pathology results

The postoperative pathological diagnosis is CCRCC (ISUP/WHO grade: 2).

Gross examination revealed a kidney with perirenal fat, measuring 18.9×9.1×7.4 cm in size. The kidney section appeared multi-cystic and solid, with cyst cavities ranging from 0.5cm to 3.8cm in diameter and wall thicknesses of 0.1cm to 0.2cm. The solid areas appeared grayish-yellow in color, with a medium to soft consistency. Most of the tissue was necrotic and hemorrhagic, and no distinct renal parenchyma was visible.

**Figure 2:**
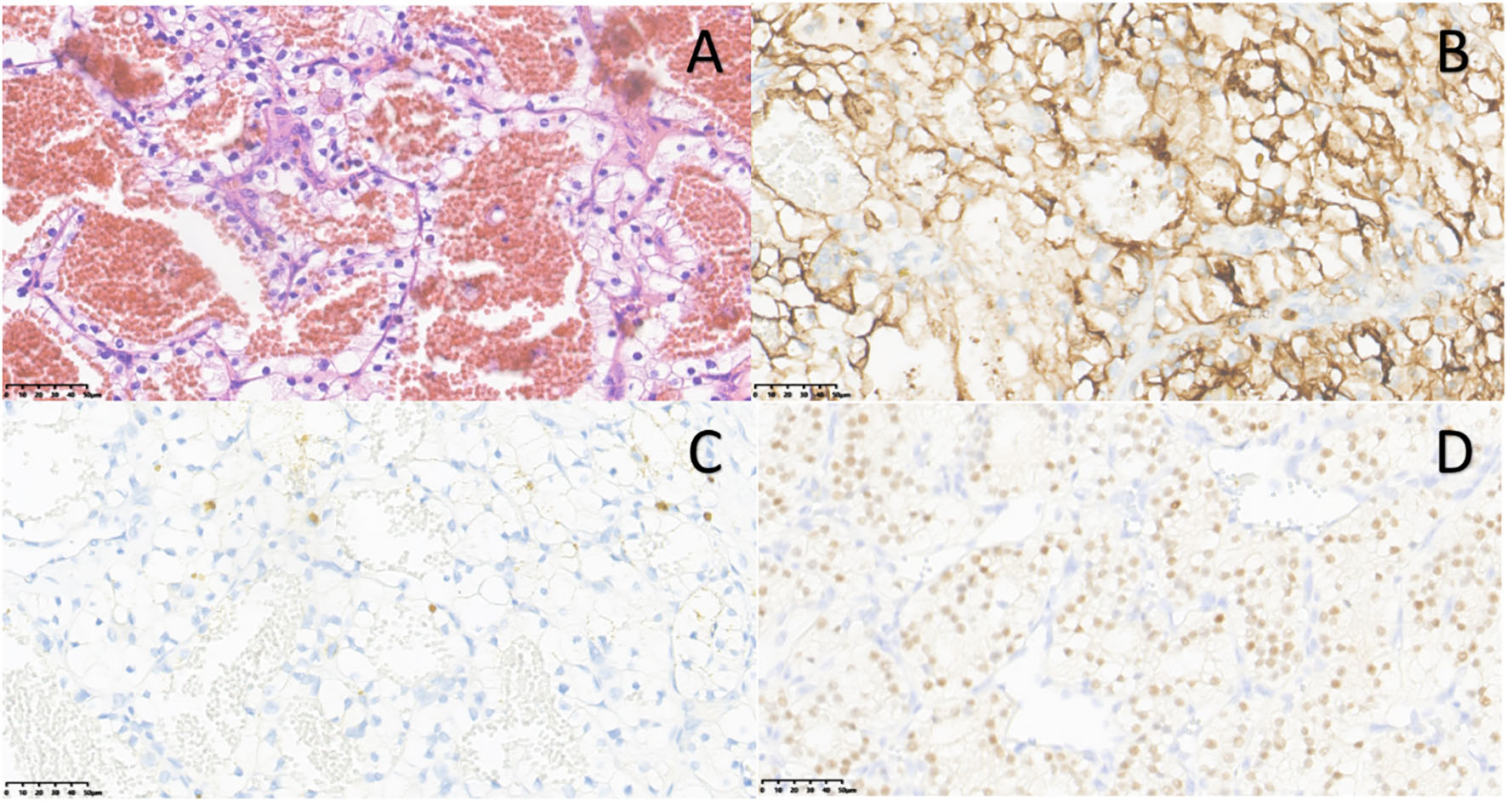
Pathological and immunohistochemistry images of left native renal carcinoma (Case 1): **(A)** Hematoxylin & eosin (H&E) staining (×40). **(B)** CD10 positive immunostaining (×40). **(C)** CK7 negative immunostaining (×40). **(D)** PAX-8 positive immunostaining (×40).

Immunohistochemistry showed that the cancer cells were positive for CAIX, CD10, RCC, Vim, PAX-8, and CD117, while negative for CK7, TFE3 (**Figure 2**).

#### Treatment intervention

A multidisciplinary diagnosis and treatment meeting was held to discuss the therapies of this case. The patient eventually underwent laparoscopic left nephrectomy under general anesthesia. Postoperative tests show serum creatinine at 255 μmol/L and uric acid at 166 μmol/L. The immunosuppressive regimen includes: Moxifloxacin hydrochloride tablets; Ganciclovir capsules; Prednisone acetate tablets; Mycophenolate mofetil; Sirolimus Capsules; and Roxadustat capsules. It is recommended that the patient return for weekly follow-ups, with adjustments to the hormone and anti-rejection medications based on the results.

### Case 2

#### Chief complaints

A 45-year-old male with a history of allogeneic renal transplantation 7 years ago, presenting with generalized rash and dyspnea for 1 week.

#### History of present illness

Seven years ago, the patient underwent allogeneic renal transplantation for ESRD. Three years post-transplant, routine follow-up revealed elevated serum creatinine (182.0 μmol/L), suggestive of chronic rejection. Intravenous immunoglobulin (IVIG) therapy was initiated, with subsequent serum creatinine levels fluctuating between 150–200 μmol/L. During this period, the patient intermittently experienced lower limb and facial edema. Two years ago, a renal biopsy was performed at our institution. Pathological diagnosis confirmed chronic active antibody-mediated rejection (CAAMR). Treatment included three sessions of plasma exchange, combined with high-dose IVIG (150 g total) and methylprednisolone pulse therapy, after which the patient was discharged with stabilized renal function. One week prior to admission, the patient developed generalized vesicles and progressive dyspnea.

#### Physical examination

The patient presented with no obvious tenderness or rebound tenderness in both kidney areas, no significant tenderness in the bilateral ureter areas, and no palpable mass in the suprapubic region.

#### Imaging examinations

**Figure 3:**
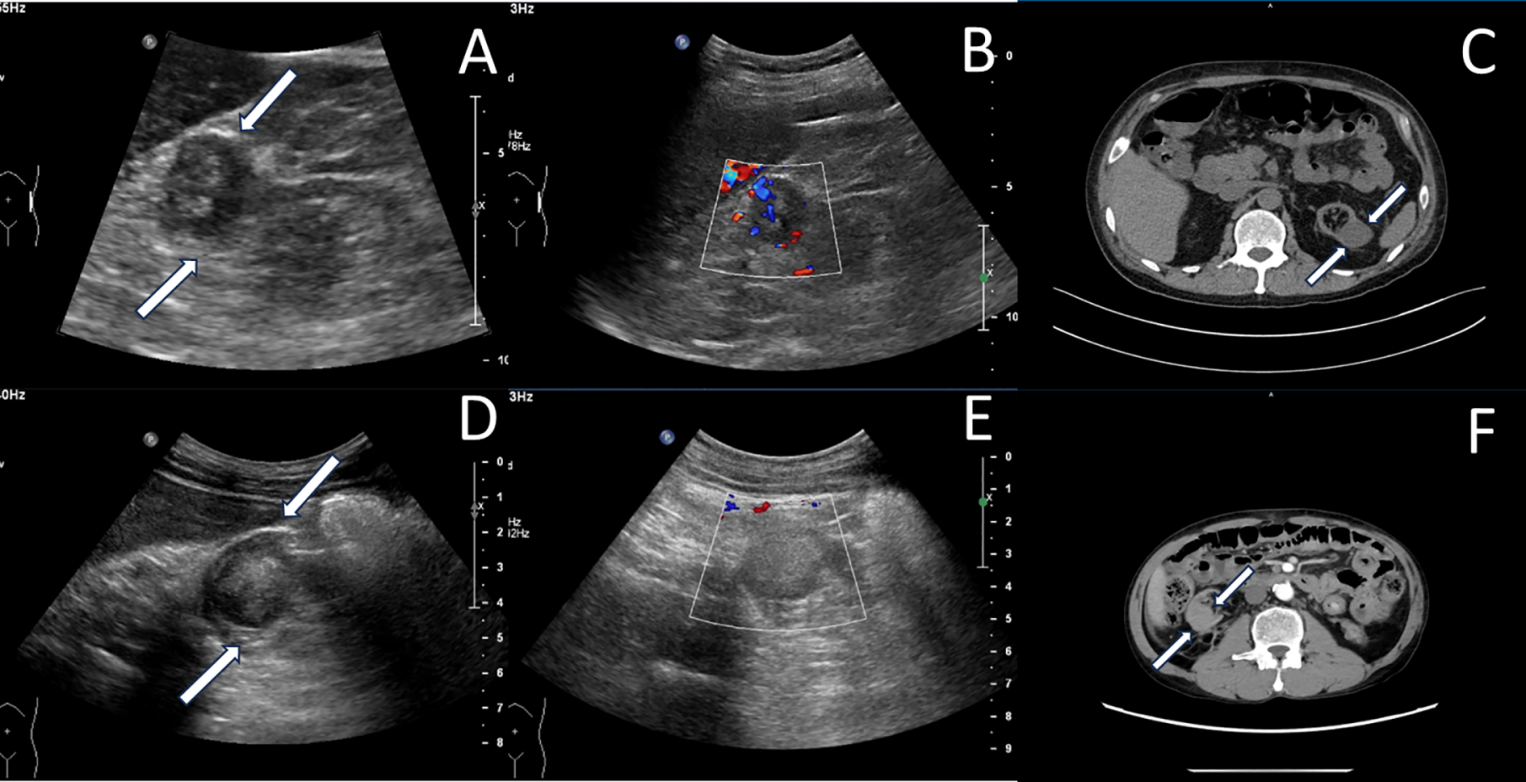
Native renal tumors in other cases. Case 2: **(A)** A hypoechoic mass(white arrow) with relatively clear boundaries was detected in the upper portion of the left native kidney. **(B)** Color Doppler shows intralesional vascularity (dot/linear signals). **(C)** Computed tomography showed a slightly low-density mass in the superior-lateral portion of the left kidney (white arrow). Case 3: **(D)** A hypoechoic mass(white arrow) was detected in the middle and lower portions of the right native kidney. **(E)** Color Doppler ultrasound showed absence of vascularity within the mass. **(F)** Enhanced computed tomography demonstrates heterogeneously enhancing mass in atrophic right kidney (white arrow).

The ultrasound examination revealed bilateral native kidneys atrophic, with increased parenchymal echogenicity and ill-defined corticomedullary junction. A 2.6 × 2.3 cm hypoechoic mass at the upper pole of the left native kidney, demonstrating well-defined margins, homogeneous echotexture, and rich internal vascularity on Doppler (**Figures 3A, B**). The ultrasound diagnosis indicated the solid renal mass suspicious for malignancy.

Enhanced CT scan revealed a 2.8 cm hypodense mass in the superior lateral left kidney with heterogeneous enhancement, highly suggestive of RCC (**Figure 3C**).

#### Pathology results

Pathological diagnosis is CCRCC (WHO/ISUP grading, nuclear grading, Grade 2, with Grade 3 in a few areas).

Gross examination revealed a nodular mass measuring 2.7 × 2.2 × 2 cm adjacent to the subcapsular region of the upper pole of the left kidney. The cross-section appeared grayish-white to grayish-yellow, solid, multicolored, with visible hemorrhage, seemingly invading the renal capsule.

Immunohistochemistry showed RCC (+), CD10 (+), CA9 (+), TFE3 (-), SDHB (+), FH (+), Pax8 (+), HMB45 (-).

#### Treatment intervention

The patient underwent laparoscopic left nephrectomy under general anesthesia.

### Case 3

#### Chief complaints

A 51-year-old male with a history of renal transplantation 15 years ago presented with a 10-month history of hematuria.

#### History of present illness

Over 15 years ago, the patient underwent allogeneic renal transplantation at an external hospital for ESRD, followed by graft failure and initiation of regular dialysis. Ten years ago, the patient received a second allogeneic renal transplantation at our institution. Three years prior to presentation, serum creatinine levels rose to over 400 μmol/L with a daily urine output of approximately 1000 mL. Suspected renal transplant rejection prompted a transplant kidney biopsy at our center, with histopathology suggestive of CAAMR. Subsequently, the patient resumed regular hemodialysis three times weekly, with an ultrafiltration volume of approximately 2000 mL per session. Urine output progressively declined to 20-30 mL daily. Ten months ago, the patient developed hematuria without apparent precipitating factors and presented to our hospital for further evaluation.

#### Physical examination

No significant clinical signs of the patient were observed.

#### Imaging examinations

Bilateral native kidneys demonstrated atrophy with increased parenchymal echogenicity and loss of corticomedullary differentiation. A well-circumscribed, hypoechoic mass (2.3×1.9×2.2 cm) was identified in the mid-to-lower pole of the right kidney, showing homogeneous echotexture and absence of internal vascularity on Doppler imaging (**Figures 3D, E**). The ultrasonic diagnosis was a solid mass in the right kidney, suspicious for malignancy pending histopathological confirmation.

Enhanced CT scan showed atrophic right native kidney containing a hyperattenuating nodule (2.5 cm in diameter) with heterogeneous enhancement, suggestive of neoplastic lesion, with the possibility of cysts with hemorrhage cannot be ruled out (**Figure 3F**).

#### Pathology results

Pathological diagnosis is PRCC (WHO/ISUP grade: 2).

Gross examination revealed a nodular mass measuring 2.8×2.5×1.4 cm in the mid-lower pole of the right kidney, exhibiting a grayish-white to yellow cut surface with areas of hemorrhage and necrosis. Tumor invasion into the renal capsule was observed.

Immunohistochemistry showed CD10 (+), CA IX (-), CK7 (+), TEF3 (-), SDHB (+), FH (+), CK (Pan) (+), CD34 (-), KSP-Cadherin (-), CK20 (-).

#### Treatment intervention

The patient underwent laparoscopic right nephrectomy under general anesthesia.

## Discussion

ESRD represents a clinical syndrome characterized by chronic, progressive, and irreversible damage to the renal parenchyma ​resulting from multiple etiologies. When the disease progresses to its terminal stage, renal function is almost completely lost. Renal transplantation remains as the preferred therapeutic option for ESRD. According to large-scale clinical studies, patients who have undergone renal transplantation exhibit a 48-82% reduction in long-term mortality compared to those remaining on the transplant waiting list (8). Additionally, transplantation eliminates the need for peritoneal dialysis or hemodialysis, thereby mitigating complications (10).

However, KTRs continue to face a significantly increased risk of malignant tumors. Notably, the incidence of RCC in native kidneys of KTRs is significantly higher than in transplanted kidneys. Importantly, RCC in native kidneys frequently presents asymptomatically, lacking classic signs such as hematuria or flank pain, which delays diagnosis and worsens prognosis. Surveillance through imaging are critical for early detection and intervention, thereby reducing morbidity and mortality in this high-risk population.

### Epidemiological, clinical, and pathological characteristics

Epidemiological studies indicate that the cumulative incidence of DPTM is 4% to 5% at 5 years, rising to 10% at 10 years, and exceeding 25% at 20 years (1, 11). Notably, the cumulative mortality rates among KTRs with DPTM are 14%, 32%, and 48% at 5, 10, and 15 years of follow-up, respectively (2).

The incidence of RCC in KTRs is significantly elevated (0.7%), representing at least a 6.8-fold increase compared to the general population. Approximately 80.4% of RCC cases occur in the native kidneys prior to transplantation (4–7). Specifically, 0.5% of KTRs develop RCC within 5 years post-transplant, increasing to 1.0% after 10 years (4). Compared to non-transplant RCC patients, KTRs exhibit significantly lower 5-year cancer-specific survival and non-recurrence rates, with rates of 79.6% and 59.2%, respectively (*p*<0.05) (12).

The elevated RCC risk in KTRs is multifactorial, including age, Epstein-Barr virus infection, chronic inflammation (elevated median C-reactive protein levels), acute graft rejection, immunosuppression (e.g., tacrolimus), and prolonged dialysis (>10 years) (2–4).

According to the 2022 World Health Organization histopathological classification criteria, RCC is a histopathologically heterogeneous malignancy with diverse subtypes (13). In non-transplant patients, CCRCC predominates (70–80% of cases), followed by PRCC (10–15%) and chromophobe renal cell carcinoma (CHRCC) (5%) (14–19). Among KTRs, while CCRCC remains most common (32–45%), the incidence of pRCC in native kidneys rises significantly (28–42%) (4, 8).

CCRCC originates from the epithelium of proximal renal tubules and is characterized by high incidence and poor prognosis, making it one of the most common subtypes of RCC. Histologically, it exhibits solid, nested, tubular, acinar, or papillary growth patterns, abundant intratumoral vasculature, and frequent hemorrhage/necrosis (20).

In contrast, PRCC and CHRCC are low-grade malignant renal tumors, typically exhibiting hypovascular features. PRCC arises from the epithelium of distal convoluted tubules in the kidney, with pathological features of papillary or small tubular arrangements of cells (21, 22). CHRCC, originating from the epithelium of renal collecting tubules, often has a central tumor location within the medulla. The tumor cells are arranged along dense fibrovascular stroma, relatively uniformly distributed, and due to the lack of abundant vascular sinuses in the tumor tissue, hemorrhage, necrosis, and cystic degeneration are less common (23).

Accurate RCC subtyping is critical for personalized therapeutic strategies and prognostic assessment, ultimately optimizing patient management.

### Imaging examination methods

Incidentally diagnosed renal masses account for 60% of newly diagnosed renal tumors, with the majority being small renal masses (SRMs) ≤4 cm (24–27). The primary imaging modalities include ultrasonography, CT, and Magnetic Resonance Imaging(MRI). Ultrasonography, due to its simplicity, non-invasiveness, and high resolution, is particularly suitable for evaluating solid or cystic renal masses in native or transplanted kidneys, making it the preferred screening method.

CEUS exhibits a median diagnostic sensitivity of 93%, offering advantages in both temporal and spatial resolution (28, 29). It accurately characterizes SRM size and enhancement patterns without exposing patients to ionizing radiation or nephrotoxic agents (**Table 1**) (30). Additionally, CEUS enables real-time monitoring of contrast agent inflow and outflow within renal lesions, providing dynamic perfusion information. This capability enhances the differential diagnosis of pseudocapsules and cystic renal lesions compared to contrast-enhanced CT (23, 31).

**Table 1:** Ultrasonographic characteristics of common renal tumors.

Entities	Conventional ultrasound characteristics	CEUS characteristics
CCRCC	Typically hypoechoic with heterogeneous echogenicity, often encapsulated by a pseudocapsule.	Heterogeneous enhancement with a ring-like enhancing margin (pseudocapsule sign). Enhancement pattern: rapid wash-in and rapid wash-out.
PRCC	Usually isoechoic with heterogeneous echogenicity.	Homogeneous enhancement. Enhancement pattern: slow wash-in and rapid/slow wash-out.
CHRCC	Homogeneous echogenicity; tends to expand toward the renal pelvis and extrarenal regions.	Slow wash-in and slow wash-out enhancement pattern.
RAML	Variable echogenicity (slightly hyperechoic or hyperechoic) depending on fat/smooth muscle content; sparse vascularity.	Homogeneous enhancement. Enhancement pattern: rapid wash-in and slow wash-out.
RO	Isoechoic or slightly hyperechoic.	More than 50% of ROs demonstrate “synchronous/slow wash-out”;one-third of ROs often exhibit central irregular non-enhancing areas.

CEUS, contrast-enhanced ultrasound; CCRCC, clear cell renal cell carcinoma; PRCC, papillary renal cell carcinoma; CHRCC, chromophobe renal cell carcinoma; RAML, renal angiomyolipoma;RO, renal oncocytoma.

#### Conventional ultrasound and contrast-enhanced ultrasound characteristics

##### Clear Cell Renal Cell Carcinoma

**Figure 4:**
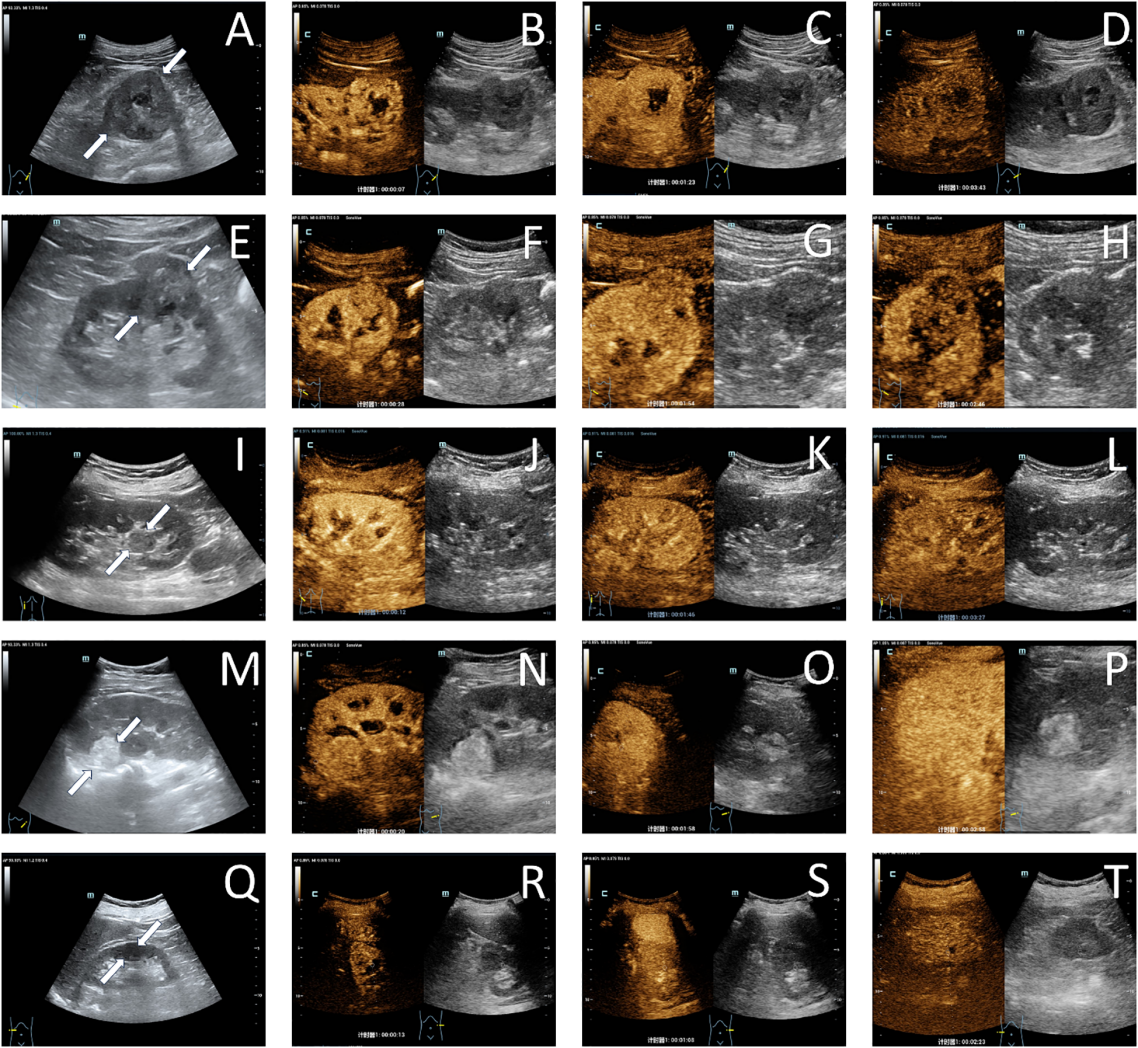
Ultrasonographic images of common renal tumors. A case of clear cell renal cell carcinoma in the left native kidney demonstrated a hypoechoic lesion (white arrow) on grayscale ultrasound imaging **(A)**. During the cortical phase **(B)**, the lesion exhibited heterogeneous hyperenhancement, followed by gradual washout in the medullary phase **(C)**, and ultimately appeared as hypoenhancement in the delayed phase **(D)**, with a non-enhancing liquefied necrotic area visible within the lesion; A case of papillary renal cell carcinoma in the right native kidney presented with a slightly hypoechoic lesion (white arrow) on grayscale ultrasound imaging **(E)**. The lesion demonstrated mild hypoenhancement during the cortical phase **(F)** and subsequently underwent slow washout, exhibiting low enhancement in both the medullary phase **(G)** and the delayed phase **(H)**; A case of chromophobe renal cell carcinoma in the left native kidney demonstrated a slightly hypoechoic lesion on grayscale ultrasound imaging, with a distinctive protrusion into the renal pelvis (white arrow) **(I)**. The lesion exhibited uniformly mild hypoenhancement during the cortical phase **(J)**, medullary phase **(K)**, and delayed phase **(L)**; A case of angiomyolipoma in the left native kidney presented with a slightly hyperechoic lesion (white arrow) on grayscale ultrasound imaging **(M)**. The lesion demonstrated isoenhancement during the cortical phase **(N)**, medullary phase **(O)**, and delayed phase **(P)**; A case of oncocytoma in the right native kidney demonstrated a hypoechoic lesion (white arrow) on grayscale ultrasound imaging **(Q)**. The lesion exhibited synchronous enhancement during the cortical phase **(R)**, medullary phase **(S)**, and delayed phase **(T)**, with a linear non-enhancing area visible in the center of the lesion.

CCRCC lesions typically appear hypoechoic with heterogeneous echotexture​ on conventional ultrasound (23, 32, 33). CEUS demonstrates a characteristic “fast-in, fast-out “ enhancement pattern with hyperenhancement​ (17, 23, 34) (**Figures 4A–D**). This dynamic contrast behavior of correlates with high microvascular density​ and formation of arteriovenous fistulas​ within tumors (23, 30, 31). Other CEUS features include: a. Heterogeneous enhancement: Predominant in lesions >3 cm, attributable to intratumoral hemorrhage, necrosis, and cystic degeneration (19, 30, 35, 36). Rapid tumor growth outstrips neovascularization capacity, leading to ischemic necrosis (30, 31); b. Pseudocapsule sign (24, 32):Observed in 62-78% of lesions measuring 2.1-5 cm (19, 27, 30). Manifests as a circumferential hyperenhancing rim​ on CEUS, resulting from ​compression-induced fibrosis​ at the tumor-normal parenchyma interface (24, 30).

##### Papillary Renal Cell Carcinoma

PRCC lesions are predominantly isoechoic with heterogeneous echotexture (23, 33). On CEUS, they demonstrate a “slow-in, fast/slow-out” enhancement pattern accompanied by low enhancement intensity (23, 34) (**Figures 4E–H**). Homogeneous enhancement becomes more frequent in PRCC when lesion diameter exceeds 3 cm (35). The low microvascular density of PRCC correlates with its characteristic hypoenhancement on CEUS, which shows high specificity for PRCC detection (27).

##### Chromophobe Renal Cell Carcinoma

Approximately 70% of CHRCC lesions exhibit homogeneous echogenicity, often displaying expansive growth patterns toward the renal pelvis and extrarenal regions on ultrasonography (31). CEUS typically reveals a “slow-in, slow-out” enhancement pattern in CHRCC (23)(**Figures 4I–L**). During the cortical phase, gradual homogeneous low enhancement is observed in CHRCC, followed by progressive contrast washout (34).

The echotexture homogeneity/heterogeneity strongly correlates with enhancement patterns, pathological characteristics, and microvascular density across RCC subtypes (23, 30, 31). Both PRCC and CHRCC exhibit slower tumor doubling times compared to CCRCC, allowing better differentiation of neovascularization within lesions.

Notably, CHRCC lesions lack abundant blood sinuses, possess sparser stromal vasculature with thicker vessel walls (21), resulting in delayed contrast agent penetration into tissue. These pathophysiological features collectively explain the predominant “slow-in, slow-out” enhancement patterns with low enhancement intensity observed in both PRCC and CHRCC on CEUS (23).

Furthermore, RCC requires differentiation from common benign renal tumors.

##### Renal Angiomyolipoma (RAML)

The sonographic and CEUS features of RAMLs correlate with their vascular, smooth muscle, and adipose components. Approximately 83.9% of RAMLs appear hyperechoic on ultrasound, with 77.4% demonstrating sparse blood flow (32, 33). CEUS typically reveals a “fast-in, slow-out” enhancement pattern, often accompanied by homogeneous enhancement (17, 30, 33, 34) (**Figures 4M–P**). Lipid-poor RAMLs exhibit higher enhancement than renal cortex during the cortical phase (27).

##### Renal Oncocytoma (RO)

About 60.9% of ROs are isoechoic on ultrasound. Over 50% display synchronous or slow wash-out on CEUS. Additionally, one-third of ROs present a non-enhancing central area with an irregular, stellate morphology on CEUS, corresponding to a central fibrotic scar (37) (**Figures 4Q–T**). This delayed wash-out pattern may result from the absence of vascular elastic fibers in tumor vessels and the lack of intralesional arteriovenous shunting (38).

#### Quantitative Ultrasound Parameter Characteristics

Quantitative ultrasound parameters also aid in further distinguishing between different pathological types of renal tumors. In the study by He et al. (30), peak enhancement intensity and wash-in area under the curve (WiAUC) values exhibited a significant gradient: CCRCC > non-CCRCC lesions. These biomarkers quantify tumor microvascular perfusion characteristics. Lu et al.’s analysis (36) revealed distinct enhancement patterns: a. homogeneous enhancement cohort: Tumor-to-cortex ratio followed CCRCCs > RAMLs > PRCCs = CHRCCs; b. heterogeneous enhancement cohort: Demonstrated CCRCCs > RAMLs = PRCCs = CHRCCs.

#### Other imaging modalities

While CT and MRI serve as primary methods for evaluating indeterminate renal masses, their diagnostic accuracy in distinguishing benign lesions (such as ROs and RAMLs) rom malignancies remains suboptimal (26).

CT demonstrates a median sensitivity of 88% and specificity of 75% in diagnosis of renal masses (29). As the first-line modality for RCC staging, multiphase contrast-enhanced CT excels in tumor localization, lymph node assessment, and detection of vascular invasion—particularly in identifying renal vein/inferior vena cava tumor thrombi and adjacent organ involvement, surpassing CEUS in these domains (23).

MRI has a median diagnostic sensitivity of 87.5% and specificity of 89% (29). Its multiplanar capabilities provide enhanced visualization of venous system infiltration, outperforming CT in assessing tumor-vascular interface (25).

Advanced functional imaging modalities show distinct clinical utility: Positron emission tomography is helpful in differentiating renal oncocytoma from CCRCC and PRCC. It is noteworthy that for differentiating between renal oncocytoma, CCRCC, and PRCC, SestaMIBI SPECT/CT displayed a specificity of 98% (95% CI, 91–100%) and a comparable sensitivity (26).

### Imaging surveillance protocol

Postoperative imaging surveillance should be systematically implemented to assess native renal status. In suspected cases or high-risk cohorts, intensified monitoring intervals with multimodal imaging integration (e.g., contrast-enhanced CT/MRI + CEUS) are warranted. The surveillance protocol must be individualized, incorporating three critical components: a. modality selection based on renal function and radiation exposure risks; b. risk-stratified frequency adjustments; c. coordinated therapeutic planning with transplant teams. Guideline-directed protocols specify baseline imaging at 3-6 months postoperatively, transitioning to annual evaluations after three disease-free years, optimizing early RCC detection and secondary prevention (16, 39).

### Treatment modalities

The treatment strategy for RCC in KTRs necessitates a comprehensive consideration of the tumor location, size, stage, and the patient’s overall health status. According to the latest guidelines from the European Association of Urology and the American Urological Association, surgical resection remains the preferred treatment option for localized RCC (16, 40). Surgical resection include partial nephrectomy (PN) and radical nephrectomy (RN). Specifically, PN is the first-line choice for localized T1-stage RCC. Among KTRs, approximately 67% of patients with native kidney RCC undergo PN, while an additional 19% receive RN (8).

However, when performing surgery in KTRs, careful consideration must be given to balancing surgical risks with preserving transplanted kidney function. Although immunosuppressants are essential for maintaining graft viability, they may compromise immune surveillance, potentially influencing tumor progression and metastasis. Therefore, immunosuppressive regimens—including dosage and agent selection—should be tailored based on individual factors such as tumor stage, immune status, and graft function.

## Conclusion

KTRs remain at an elevated risk for developing RCC within their native kidneys, with clinical manifestations that are frequently subtle or non-specific. The cases presented here underscore the significant risk posed by RCC, particularly in patients who omit regular imaging surveillance. Current evidence strongly supports the critical role of periodic imaging in early RCC detection, directly correlating with improved survival rates and prognosis in KTR populations. Notably, ultrasonography and CEUS have become indispensable diagnostic tools due to their non-invasive nature, high-resolution capabilities, and real-time dynamic assessment of renal lesions. Implementation of regular follow-up protocols stratified by individual risk profiles is essential to enable timely interventions. While surgical resection remains the gold-standard treatment for localized RCC, surgeons must meticulously balance operative risks against the imperative to preserve transplanted kidney function. Collectively, adherence to standardized imaging surveillance protocols combined with prompt therapeutic strategies constitutes the cornerstone for optimizing both survival outcomes and quality of life in KTRs.

## Data Availability

The original contributions presented in the study are included in the article/supplementary material. Further inquiries can be directed to the corresponding author/s.
